# Investigation and Validation of a Shape Memory Alloy Material Model Using Interactive Fibre Rubber Composites

**DOI:** 10.3390/ma17051163

**Published:** 2024-03-01

**Authors:** Achyuth Ram Annadata, Aline Iobana Acevedo-Velazquez, Lucas A. Woodworth, Thomas Gereke, Michael Kaliske, Klaus Röbenack, Chokri Cherif

**Affiliations:** 1 Institute of Textile Machinery and High Performance Material Technology, TU Dresden, 01062 Dresden, Germany; thomas.gereke@tu-dresden.de (T.G.); chokri.cherif@tu-dresden.de (C.C.); 2 Institute of Control Theory, Faculty of Electrical and Computer Engineering, TU Dresden, 01062 Dresden, Germany; aline_iobana.acevedo_velazquez@tu-dresden.de (A.I.A.-V.); klaus.roebenack@tu-dresden.de (K.R.); 3 Institute for Structural Analysis, TU Dresden, 01062 Dresden, Germany; lucas.woodworth@tu-dresden.de (L.A.W.); michael.kaliske@tu-dresden.de (M.K.)

**Keywords:** interactive fibre rubber composites, shape memory alloy, Woodworth and Kaliske SMA model

## Abstract

The growing demand for intelligent systems with improved human-machine interactions has created an opportunity to develop adaptive bending structures. Interactive fibre rubber composites (IFRCs) are created using smart materials as actuators to obtain any desired application using fibre-reinforced elastomer. Shape memory alloys (SMAs) play a prominent role in the smart material family and are being used for various applications. Their diverse applications are intended for commercial and research purposes, and the need to model and analyse these application-based structures to achieve their maximum potential is of utmost importance. Many material models have been developed to characterise the behaviour of SMAs. However, there are very few commercially developed finite element models that can predict their behaviour. One such model is the Souza and Auricchio (SA) SMA material model incorporated in ANSYS, with the ability to solve for both shape memory effect (SME) and superelasticity (SE) but with a limitation of considering pre-stretch for irregularly shaped geometries. In order to address this gap, Woodworth and Kaliske (WK) developed a phenomenological constitutive SMA material model, offering the flexibility to apply pre-stretches for SMA wires with irregular profiles. This study investigates the WK SMA material model, utilizing deformations observed in IFRC structures as a reference and validating them against simulated models using the SA SMA material model. This validation process is crucial in ensuring the reliability and accuracy of the WK model, thus enhancing confidence in its application for predictive analysis in SMA-based systems.

## 1. Introduction

Shape memory alloys (SMAs) have a unique ability to retain their original shape after undergoing rigorous deformations. They belong to a family of smart materials which are capable of exhibiting the shape memory effect (SME) with a wide range of applications, such as in the fields of aerospace/aircraft [1,2], robotics [3], biomedical [4], etc. SMAs have high energy density, which corresponds to high actuation forces, and they are highly capable of recovering their original shape due to phase transformations triggered by temperature differences and also by the applied stress field [5]. During the phase transformations, structural changes happen due to shear lattice distortions, which allow the SMA to transform from one phase to another during different temperatures and stress-inducing conditions. SMAs have two phases, namely, austenite and martensite. Martensite is formed by the forward transformation upon cooling from austenite, and austenite is formed upon heating from martensite. Martensitic crystals contribute to many martensitic variants and are further classified into twinned and detwinned martensite structures. The phase changes between austenite and twinned and detwinned martensite correspond to the prolific properties of SME and super-elasticity (SE). SME is further classified into one-way SME (OWSME) and two-way SME (TWSME) [2,6,7].

### 1.1. SMA-Integrated Composites

The discovery of the thermoelastic effect and pseudo-elasticity in SMAs paved the way for many revolutionary industrial applications. Nickel-titanium alloy (Nitinol) became a centre of attraction due to its properties, such as corrosion resistance, wear resistance, bio-compatibility, and low-cost production compared to other SMAs, which helped toward achieving a commercial breakthrough in the 1990s with the manufacturing of biomedical stents [8]. The functional properties of Nitinol in the field of bio-medics have extended further into orthodontics, orthopaedics, vascular applications, etc. [4,9]. The operating temperatures of Nitinol advanced the use of SMAs in transport applications, such as cars and aircraft, leading to improved efficiency due to cost and weight reduction. Their unique properties are also being utilized in the aerospace sector, such as with morphing concepts [10,11], vibration dampers, release mechanisms, etc. SMAs have a considerable amount of recoverable strains. This spiked interest in the research community to use SMAs with soft elastomer and bio-inspired materials, which contribute to better flexibility when compared with conventional actuators [12]. There have been many developments pertaining to the use of SMA in robotics. Many actuator concepts have been developed in the last two decades, involving SMA as the driving force in the field of soft robotics. Some interesting actuator concepts include crawler robots [13], jumper robots [14], flower robots [15], fish robots [16,17,18], locomotion robots [19], bio-mimetic robotic hand [20], soft robotic tendon grippers [12,21], etc.

### 1.2. SMA Material Models

Simulating the behaviour of shape memory alloys SMAs plays a critical role in advancing and unlocking their potential across diverse applications by aiding in the understanding their intricate behaviour under varying conditions. They allow the user to optimize the design of several engineering applications, such as actuators, sensors, and medical devices, to identify their behaviour in different operating conditions. Such applications also require the extensive testing of SMAs, which proves to be costly and time-consuming. Simulation offers multiple pathways, including design optimization and parameter studies, to streamline extensive testing by generating a wide range of possibilities. This approach also enables researchers to gain insights into the performance of SMA-based systems. The necessity to predict the structural integrity of SMA-driven applications serves as a basis for developing various mathematical models to understand the systematic behaviour of SMAs.

By combining the laws of thermodynamics with solid-solid phase transformations in terms of free energy and dissipation potential, Fremond [22] proposed a mathematical model for the macroscopic thermo-mechanical evolution of SMAs. In this model, temperature and displacements are unknown variables that are coupled to the partial differential inclusion of martensite and austenite phase variables. Colli et al. [23], on the basis of this model, addressed the one-dimensional case of asymptotic stability for dynamical systems and have received higher considerations pertaining to the qualitative behaviour of systems together with the Falk-Konopka model [24], in which the free energy of the crystal structure relies upon the full-strain tensor and temperature. Colli et al. [25] also proved that the assumption of considering the positive temperature in the Fremond model is, in fact, attained everywhere in the system. There are many other thermo-mechanical models, for example [26,27,28,29,30], explaining the behaviour of SMAs with respect to martensitic deformations and their implementations into finite element analysis (FEA). The Souza and Auricchio (SA) [31,32,33] model is one of the models that is implemented in the finite element method (FEM) software ANSYS (https://www.ansys.com/). This approach takes both the effects of thermal and mechanical loading into account and is considered one of the prominent models in which a constitutive model is designed to describe the behaviour of SMAs by considering the energy and dissipation mechanisms, with a tensor variable representing the inelastic strain. This allowed researchers to add and incorporate new features and phenomena into the model.

**Figure 1:**
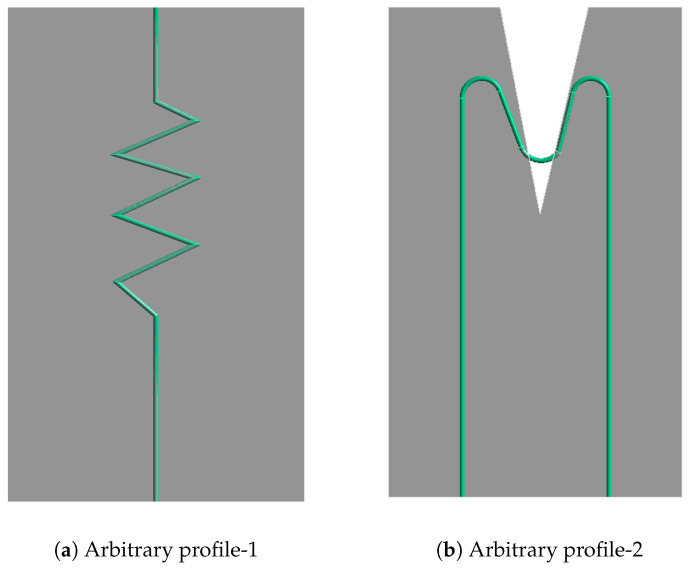
Arbitrary SMA profile models embedded in silicone.

One limitation of this model is that, from the geometrical point of view, it is very difficult to produce pre-stretch in bent geometries and further bring them into the exact required shape that is present in the composite. In soft structural applications, traditional methods, such as housing or clamping, are not feasible due to their added weight, which compromises structural efficiency. In order to overcome this limitation, textile technologies can be employed during the manufacturing process to integrate SMA wires through stitching. This method ensures that both wire ends are closely positioned, allowing for fixation at just one end of the soft structure, resulting in a non-linear SMA wire that necessitates pre-stretching in computational simulations for accurate behaviour prediction. However, this constraint limits the efficacy of predicting deformations in more diverse SMA geometries found in broader applications. For visualization, consider a simulation model with arbitrary SMA wire profiles embedded in silicone, as shown in Figure 1. Consider the whole model is fixed at the bottom. In both profiles, it is not feasible to pre-stretch the complete wire by applying force or displacement boundary conditions to induce the strain required to obtain deformation in the body using the SA model due to the wire design.

**Figure 2:**
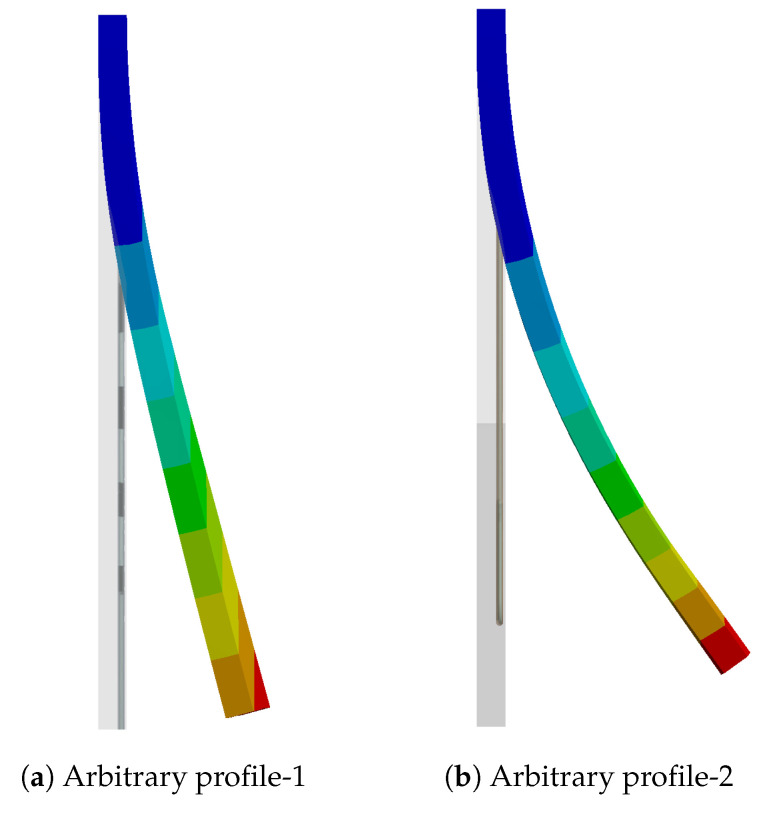
Deformation observed on the arbitrary profiles.

In order to overcome this, Woodworth et al. [34,35] proposed a material model (the Woodworth and Kaliske (WK) model) based on the SA model formulation by incorporating functional fatigue (FF) and transformation-induced plasticity (TRIP), along with the consideration of pre-stretch in the SMA wires. This model was initially developed to determine the cyclic behaviour of SMAs and was extended for pre-stretching, which allows for the modelling of different SMA wire profiles. Using the pre-stretch part of this model on the above two arbitrary SMA profiles would give the respective deformation for the body, as shown in Figure 2, by applying only temperature as the boundary condition. In order to ensure suitability for a wide range of applications, it is essential to validate the pre-stretch component of the model using various SMA profiles. In the context of this article, we utilized a U-profile SMA wire incorporated into the composite to validate the WK model. To our knowledge, the limitation regarding the application of pre-stretch to irregular geometries has not been specifically addressed in any other literature. While pre-stretch has been examined in previous studies [36,37,38], it has typically been in simplified models and for straight configurations.

In order to assess the computational efficiency and predictive accuracy of both the SA and WK models, a 1D calibration of the two models is conducted at the beginning of the results section, along with the consideration of simulation of straight SMA wire integrated IFRC models.

### 1.3. Objectives

The objective of this work is to investigate the WK model by validating it against the SA model in terms of deformations obtained in the IFRC using straight SMA wires. Moreover, the results were evaluated based on the deformations obtained in the IFRC structures, and the importance of adding pre-stretch in the model is discussed by modelling and validating a nonstraight (a U-profile) SMA wire-integrated IFRC structure.

In the next section, individual information about the components used and the methods to manufacture the IFRC are discussed along with the important aspects of both the models, which is followed by simulation and experimental results in Section 3 and discussion and conclusion in Section 4 and Section 5, respectively.

## 2. Materials and Methods

### 2.1. Materials

The SMA wire used in this work is a Nitinol wire that is 0.3 mm in diameter provided by SAES Getters (Milan, Italy). The SMA wire is obtained in a pre-strained state and is able to achieve OWSME without applying any prior external load. A twill-woven fabric made of glass fibres is used as reinforcement, with polydimethylsiloxane (PDMS also known as Sylgard 184™) supplied by Dow Corning (Midland, MI, United States) as the matrix material. Polyamide (Nylon 66) yarns supplied by Barnet Europe W. Barnet GmbH & Co. KG (Aachen, Germany) are used to braid the SMA wires [39,40] to prevent direct contact between SMA and the silicone matrix. The properties and characterization of all the materials are carried out and are well documented in [41].

**Figure 3:**
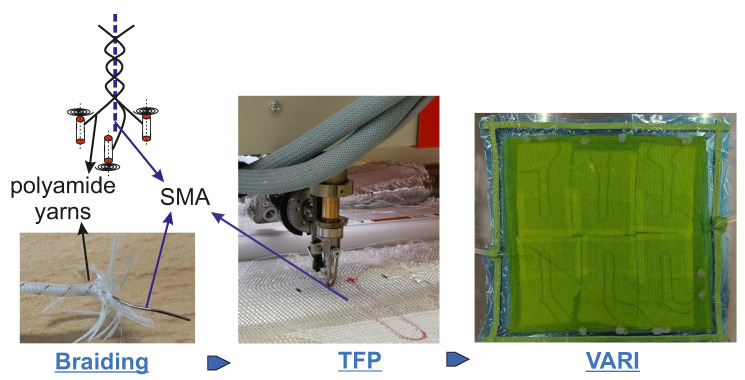
IFRC manufacturing process [44]—Braiding—TFP (Tailored Fibre Placement)—VARI (vacuum-assisted resin infusion).

The braided SMA is stitched onto the woven fabric with the help of tailored fibre placement (TFP) [42,43] technology (ZSK Embroidery Machines GmbH, Krefeld, Germany). The semi-finished composite is then infiltrated with PDMS using the vacuum-assisted resin infusion (VARI) process. The manufacturing process of the IFRC is explicitly displayed in Figure 3.

### 2.2. Souza and Auricchio Model

The Souza and Auricchio (SA) model describes the behaviour of the SMAs based on small strain theory and thermodynamic irreversible processes. The effects of thermal and mechanical loadings are considered, and the corresponding energy and the dissipation mechanism are used to evaluate the material state of the SMA. The constitutive equations in this model relate the state variables to the strain and stress of the material, and the phase structure is defined by the tensor variable representing the inelastic strain [33]. This model considers the evolution of the transformation strain during deformation and is used to account for the material’s microstructural changes due to temperature and stress.

The Helmholtz free-energy function of the SME model implemented in ANSYS is based on the 3D thermomechanical model for stress-induced solid transformations [31,32] and is given as

(1) $$\begin{array}{c} \Psi \left(\varepsilon ,T,\varepsilon_{tr}\right)=\frac{1}{2}\left(\varepsilon -\varepsilon_{tr}\right):D:\left(\varepsilon -\varepsilon_{tr}\right)+\tau_{M}\left(T\right)\left|\right|\varepsilon_{tr}\left|\right|+\frac{1}{2}h||\varepsilon_{tr}{\left|\right|}^{2}+I_{\varepsilon_{tr}}\left(\varepsilon_{tr}\right), \end{array}$$

where *D* is the material elastic stiffness tensor, ε is the total strain, $\varepsilon_{tr}$ is the transformation strain, τ(T) is a positively monotonic increasing function of temperature *T*, *h* is a hardening parameter, and $I_{\varepsilon_{tr}}\left(\varepsilon_{tr}\right)$ is an indicator function to satisfy the constraints on the transformation norm.

The stress and consistent tangent stiffness matrix are updated using the backward Euler integration scheme in the finite element analysis.

### 2.3. Woodworth and Kaliske Model

The Woodworth and Kaliske (WK) model [34] is a phenomenological mathematical model used to describe the behaviour of SMAs under mechanical loading. This model was developed considering functional fatigue (FF) and transformation-induced plasticity (TRIP) in SMAs, which can affect their response to cyclic loading. The phase transformations are often followed by the deterioration of material properties, leading to FF and the development of irrecoverable strains. A continuation of this model is developed by considering the reorientation, which contributes to the FF behaviour, the logarithmic evolution of saturation, and a pre-stretch [35]. The mechanical behaviour of the SMA model relies on a set of internal state variables, which evolve with time as a function of applied strain and temperature.

The total strain in the model is decomposed into

(2) $$\begin{array}{c} \varepsilon =\varepsilon_{e}+\varepsilon_{\theta }+\varepsilon_{t}+\varepsilon_{r}-\varepsilon_{0}, \end{array}$$

where $\varepsilon_{e}$ is the elastic strain, $\varepsilon_{\theta }$ is the thermal strain, $\varepsilon_{t}$ is the transformation strain, $\varepsilon_{r}$ is the residual or transformation-induced plasticity strain, and $\varepsilon_{0}$ is the initial strain due to pre-stretching. The initial pre-stretching in the manuscript is defined by

(3) $$\begin{array}{c} \varepsilon_{0}=\varepsilon_{L}\chi_{0}^{S}N_{t,0}, \end{array}$$

where $\varepsilon_{L}$ is the maximum transformation strain, $\chi_{0}^{S}$ is the initial-oriented martensite volume fraction, and $N_{t,0}$ is the pre-stretch transformation strain direction. These quantities are also applied as the initial conditions of the martensite volume fraction, $\chi^{S}$, and the transformation strain direction, $N_{t}$.

The total Helmholtz free energy $\psi =\psi (\varepsilon ,\theta ,\chi^{S},N_{t},\chi_{r},\chi_{d},\varepsilon_{r},B)$ is defined as

(4) $$\begin{array}{c} \psi =elastic+thermal+interaction+constraint+residual\;stress, \end{array}$$

where

(5) $$\begin{array}{c} \mathrm{elastic}\to \;\frac{1}{2}Ktr{\left(\varepsilon \right)}^{2}+\mu ||e_{e}{\left|\right|}^{2}-3\alpha Ktr\left(\varepsilon \right)\left(\theta -\theta_{0}\right), \end{array}$$

(6) $$\begin{array}{c} \mathrm{thermal}\to \;e_{0}^{A}-\eta_{0}^{A}\langle \theta -\theta_{0}\rangle +c_{v}\left[\left(\theta -\theta_{0}\right)-\theta \ln\left(\frac{\theta }{\theta_{0}}\right)\right]+▵\eta_{t}\chi^{S}\langle \theta -\theta_{0}\rangle , \end{array}$$

(7) $$\begin{array}{c} \mathrm{interaction}\to \;g^{t}\left(\chi^{S},\chi_{r},\chi_{d}\right)+\frac{1}{2}\mu_{t}\left|\right|\varepsilon_{t}{\left|\right|}^{2}, \end{array}$$

(8) $$\begin{array}{c} \mathrm{constraint}\to \;-\zeta^{S0}\left(\chi^{S}-\chi_{r}\right)-\zeta^{S1}\left(1-\chi^{S}-\chi_{d}\right)+\frac{1}{2}\zeta^{cd}\left|\right|N_{t}{\left|\right|}^{2}, \end{array}$$

(9) $$\begin{array}{c} \mathrm{residual}\mathrm{stress}\to \;-B:\varepsilon_{t}-\frac{1}{2}\varepsilon_{L}{\left||B|\right|}^{2}, \end{array}$$

where *K* is the bulk modulus, μ is the shear modulus, α is the coefficient of thermal expansion, $e_{e}$ is the deviatoric elastic strain, $e_{0}^{A}$ is the reference internal energy, $\eta_{0}^{A}$ is the reference internal entropy, $c_{v}$ is the specific heat, $▵\eta_{t}$ is entropy difference during transformation, $g^{t}$ is interaction energy due to hardening, $\mu_{t}$ is the hardening modulus for transformation, $\zeta^{S0},\zeta^{S1},\zeta^{cd}$ are Lagrange multipliers, and B is the residual stress. The constitutive equations and the discrete SMA model are elaborated in detail in the original manuscript, and it is referred to in [35].

### 2.4. Material Models for Elastomer and Fibres

PDMS was used as the elastomer in this work. An isotropic hyperelastic model by Yeoh et al. [45] was used to model the rubber. The strain energy potential used in this model is as follows:

(10) $$\begin{array}{c} W=\sum_{i=1}^{N}C_{i0}{\left(\bar{I_{1}}-3\right)}^{i})+\sum_{k=1}^{N}\frac{1}{d_{k}}{\left(J-1\right)}^{2k}, \end{array}$$

where *W* is the strain energy potential, $\bar{I_{1}}$ is the first deviatoric strain invariant, *J* is the elastic deformation gradient determinant, *N*, $C_{io},$ and $d_{k}$ are the material constants, with the third order polynomial N=3, the constants $C_{10},C_{20},C_{30},d_{1},d_{2},d_{3}$ are to be obtained by the curve fitting of the model.

The glass fibres (GFs) used in this work have a fineness of 272 Tex and were assigned to an orthotropic elastic material model with the elastic modulus in the main fibre direction, and the second and third directions were defined for numerical stability. The model parameters used for this work are considered from the previous works in the line of project from Lohse et al. [41,46] and are shown in Table 1 and Table 2.

### 2.5. Modelling Approach

In this paper, the modelling approaches for IFRC structures are compared based on the SA and WK models. The comparison is made to validate the user-defined model and the SA model based on the deformations obtained in the IFRC structures. Moreover, the deformations of a U-profile SMA are compared using experiments to validate the pre-stretch.

#### 2.5.1. Souza and Auricchio Model

**Figure 4:**
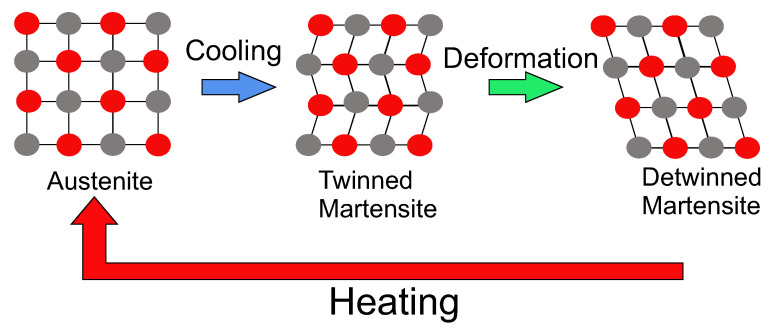
Principle of one-way shape memory effect (OWSME), illustrating the deformations within the crystal structure of SMAs [47] (edited).

ANSYS accommodates both the SE effect and the OWSME based on the formulation of the SA model. For the functionality of the IFRC structures, SME precedes the SE effect, as the deformations in the IFRC are obtained based on temperature difference (obtained via Joule heating). The principle of OWSME is such that, upon loading, a phase change is observed from twinned martensite to detwinned martensite, and, upon heating, the SMA transforms into its parent phase austenite. During this transformation, shape change is observed, and the molecular deformations are depicted in Figure 4. Upon cooling, the SMA transforms back into the twinned martensite phase without any noticeable shape change. The transformation from detwinned martensite to austenite is associated with a shape change and is obtained by heating the material at above its martensitic transformation temperature. During this process, the SMA recovers the strain induced by the loading in the martensite phase. The same principle is utilized in ANSYS to obtain the deformations in the IFRC structures.

**Figure 5:**
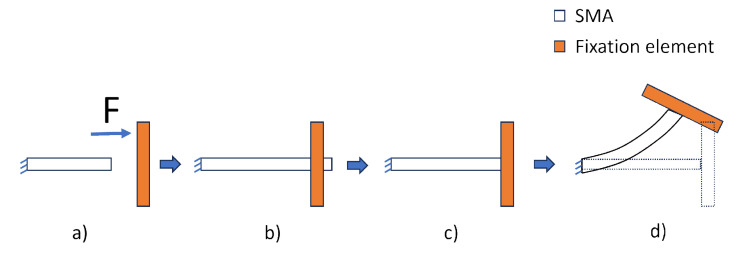
Implementation of the OWSME for IFRC modelling in ANSYS using the SA model. (**a**) → (**b**): force applied to induce strain, (**b**) → (**c**): relaxation due to elastic setback, (**c**): bonding of SMA to fixation element, (**c**) → (**d**): heating of SMA resulting in deformation.

In simulating an IFRC structure with the SA model, four load steps were considered. In the first load step, force is applied on one face of the SMA and by fixing the other side. This induces deformation due to elasticity and phase transformation in the SMA. In the second load step, force is removed to observe the elastic setback in the SMA. In the third load step, contact step control is activated to fix the SMA to the fixation element, along with the heating of the SMA in the fourth load step. This process is also shown in Figure 5. It is also worth mentioning that the third and fourth load step can be combined to reduce the computation time, but experience has shown that it is best to divide the load steps, as to ensure proper contact between the two bodies. The parameters considered for the simulation using the SA model are tabulated in Table 3.

#### 2.5.2. Woodworth and Kaliske Model

The Woodworth and Kaliske SMA model is developed and implemented in ANSYS as a user-defined function (UDF). The pre-stretch implemented in the model allows for assigning a strain in the SMA before integrating it into the composite. This allows the user to model various SMA profiles, by which the deformations can be predicted based on various arrangements of the SMA, and this reduces the modelling effort when compared to the SA model. In the experiments, the SMA wire is already in a pre-stretched state, and thus, the same length of the SMA wire can be considered in the WK model. This allows the experimental procedure to be replicated.

**Figure 6:**
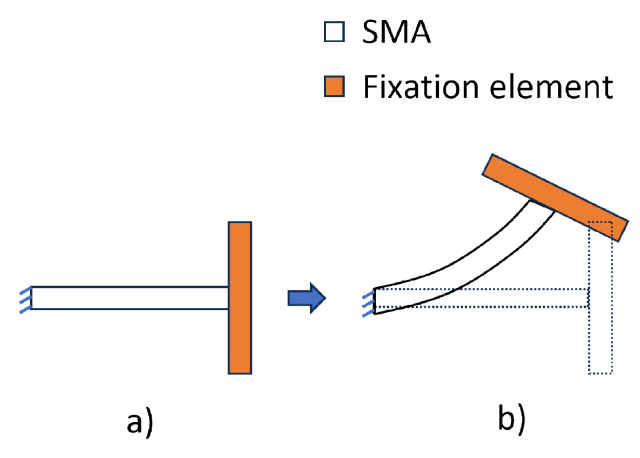
Implementation of OWSME for IFRC modelling in ANSYS using the WK model. (**a**) → (**b**): heating of SMA, resulting in deformation.

**Figure 7:**
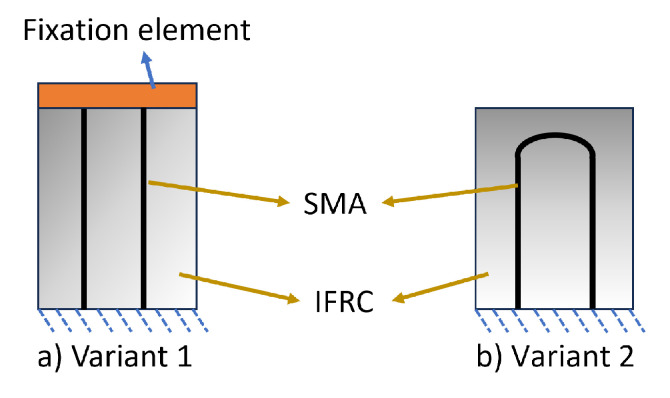
Simulation model variants for the WK model: (**a**) Variant 1: straight SMA with a fixation element at one end of SMA; (**b**) Variant 2: U-profile SMA.

In this model, temperature is considered to be the only load step that is given to the SMA wire. The SMA wire, along with the composite, is bonded with the fixation element from the beginning, as shown in Figure 6. Two SMA profiles are considered in this modelling approach, as shown in Figure 7. Variant 1 is the same as the straight SMA profile used in the SA model to compare the deformations and verify the WK model. A U-profile SMA—variant 2— is considered to validate the pre-stretch in the WK model with that of the experiments with exact geometrical structures. Some noteworthy parameters for SMA in the WK model are tabulated in Table 4. The parameters for FF and TRIP were neglected by setting them to zero in the model.

The following considerations were taken into account while modelling the IFRC structures for both models:

- A polytetrafluoroethylene (PTFE) tube with an inner diameter of 0.4 mm and an outer diameter of 0.5 mm was used in the simulation in place of the braided yarns. This assumption is made because the sole purpose of the braid around the SMA is to have indirect contact between the SMA and the elastomer. It is assumed that the effects of braiding and the braiding yarns are negligible.
- Since the targeted deformation bends, the modelling of weft yarns is neglected as they have much less influence during bending deformations [41].
- Cooling and cyclic effects are not considered in order to reduce the computational effort.
- Symmetry boundary condition in the simulation model was considered for both the variants.
- The “U” bend for the SA model was neglected, as it is not feasible to pre-strain the SMA wire, and although the WK model offers an initial pre-stretch, straight SMA wires were still modelled to validate the WK model approach against that of the SA model.
- The free end of the straight SMAs was bonded with a fixation element (polyethylene body), as shown in Figure 7a.

### 2.6. Experimental Approach—SMA Activation

**Figure 8:**
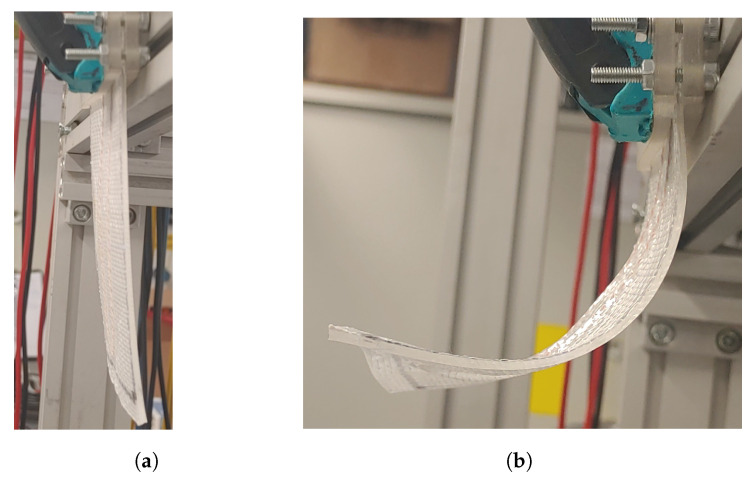
IFRC (**a**) before activation and (**b**) after activation.

The manufacturing process of the SMA wire integrated composite is explained in Figure 3. In order to activate the IFRC structures, the SMA wire was subjected to the joule effect, where electrical energy is converted into thermal energy, thereby heating the SMA wire. Upon heating, the SMA transforms into austenite, resulting in the recovery of the strain induced by pre-stretching. As the SMA wire is integrated into the composite, the strain recovery generates a force that pulls the composite in the axial direction, causing bending deformation, as depicted in Figure 8.

**Figure 9:**
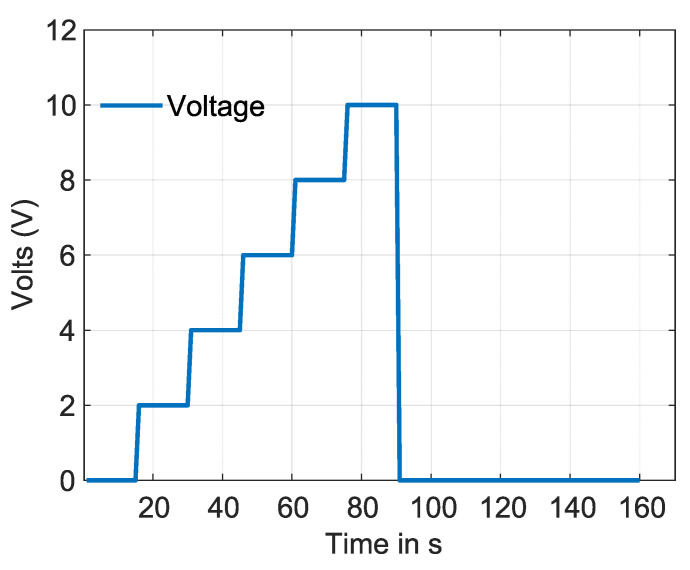
Voltage vs. time, applied to the SMA wire in the experiment.

The SMA wire is activated using different step signals that change over a specific period. This activation process can be achieved using an Arduino controlled by a Matlab script, which sends the necessary pulse-width modulation (PWM) signals. A circuit based on power metal-oxide-semiconductor field-effect transistors (MOSFETs) were utilized for this purpose. The SMA wire is activated using a PWM signal that gradually increases every 15 seconds by 20% until it reaches a maximum voltage of 10 V, with a current of 2 A. Once the voltage reaches 10 V, it is cut off to deactivate the SMA wire. The graph depicting the relationship between voltage and time is shown in Figure 9. An experimental computer vision setup was implemented to measure the deformation angle. The setup includes a computer, an Intel RealSense camera, and two-point references located at the corners of the lateral profile of the IFRC. In order to calculate the deformation angle with the camera, we identify the area of the reference points, and then the centroid of each point is calculated with the given co-ordinates. Finally, the angle θ is computed with the co-ordinates of the centroid.

An attempt was made to compare the straight SMA model results with the straight SMA experimental results by overhanging the “U” curvature part outside the composite. This experiment resulted in very low and incomparable deformation angles compared to the simulation results, and are not documented here.

## 3. Results

**Figure 10:**
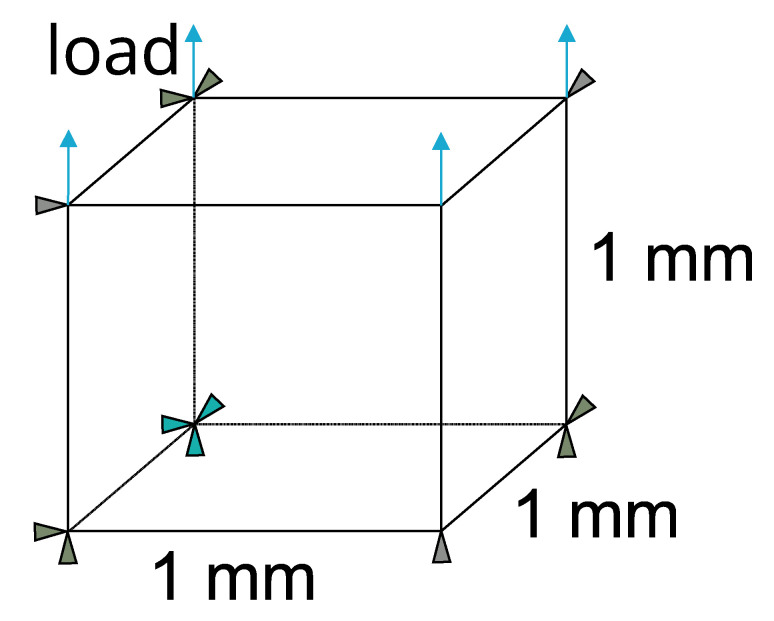
Boundary conditions for one-element model subjected to uniaxial tests.

**Figure 11:**
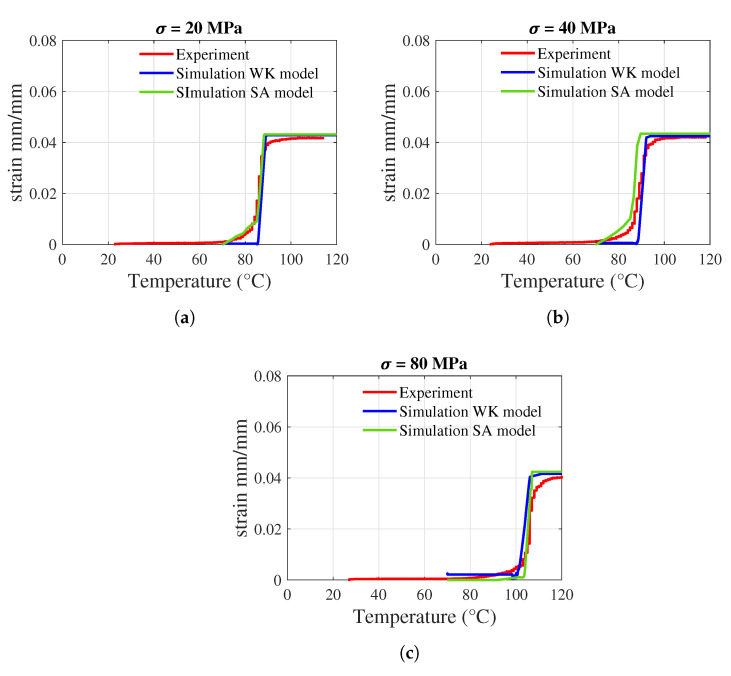
One-dimension calibration—Simulation results compared to the experimental results based on temperature-strain diagram. (**a**) 20 MPa; (**b**) at 40 MPa; (**c**) at 80 MPa.

In this section, the results of the simulations and the experiments are presented. The two material models were calibrated based on the temperature-strain graphs obtained from both simulations and experiments. The temperature-strain graph from the experiment was obtained from the uniaxial pre-strain free recovery (UPFR) test [41,48]. The material models are calibrated by considering a one-element model based on three uniaxial load cases in terms of 20 MPa, 40 MPa, and 80 MPa. The boundary conditions for the one-element model are shown in Figure 10. The cooling of SMA was not considered for both simulations and experiments. The simulation results show a general fit in the case of 20 MPa (Figure 11a), and a slight deviation is observed in the load cases 40 MPa (Figure 11b) and 80 MPa (Figure 11c). For further calibration of the WK model based on isothermal and cyclic testing, the reader is advised to refer to [34,35]. The simulation results of the SA and WK models for straight SMAs are presented, along with the simulation result for the U-profile SMA using the WK model. Then, the results from the IFRC structure activation test are presented, emphasizing the deformation angle, followed by a discussion of the results.

**Figure 12:**
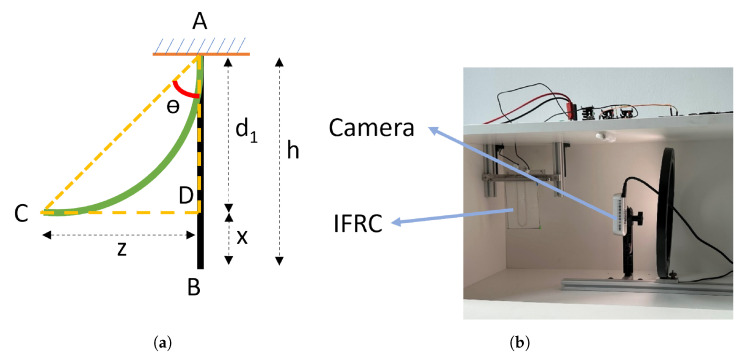
(**a**) Deformation angle θ considered for validating the bending deformations and (**b**) experimental test setup.

The simulation results for both models were cross-validated by comparing the total deformations and the volume fraction of martensite to austenite. The deformation angles were calculated numerically, taking into account the directional deformations in the models. This calculation enables the recreation of a right-angled triangle between two ends of the composite, as depicted in Figure 12a. In the experimental setup, the deformation angles are evaluated using the test setup illustrated in Figure 12b.

### 3.1. Simulation

**Figure 13:**
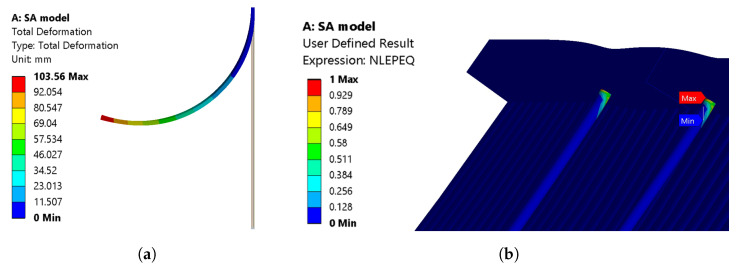
(**a**) Total deformation and (**b**) volume fraction—martensite (red colour) to austenite (blue colour) for the SA model.

**Figure 14:**
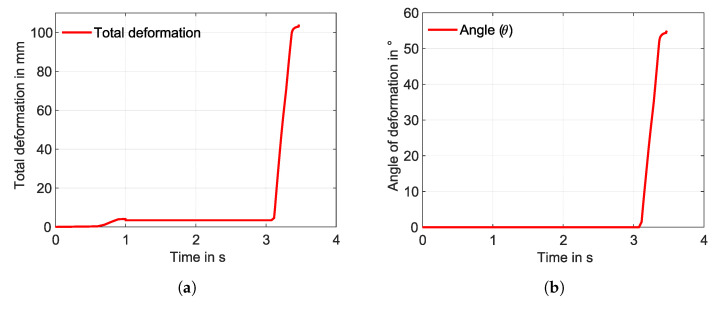
(**a**) Total deformation and (**b**) angle of deformation with respect to the time—SA model, straight SMA (simulation).

The IFRC simulation models showed the expected bending deformations. The SA model is implemented in ANSYS 2022 R1 using the procedure explained in Figure 5. A total bending deformation of 103.56 mm (Figure 13a), with a deformation angle of 55.65°, is observed. The deformation angle is obtained based on the directional deformations and the distances from the reference positions considered, as shown in Figure 12a. The simulation did not converge at the end of the load step; rather, it stopped at around 96 °C. Upon observation, the martensite to austenite transformation was complete and can be seen in Figure 13b. In the label for Figure 13b, the colour blue indicates complete transformation into the austenite, whereas red indicates complete martensite volume fraction. The corresponding graphs for total deformation and angle of deformation are shown in Figure 14a and Figure 14b, respectively.

**Figure 15:**
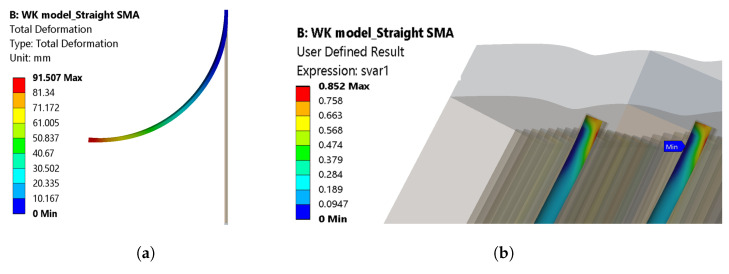
(**a**) Total deformation and (**b**) volume fraction martensite (red colour) to austenite (blue colour) for the WK model.

**Figure 16:**
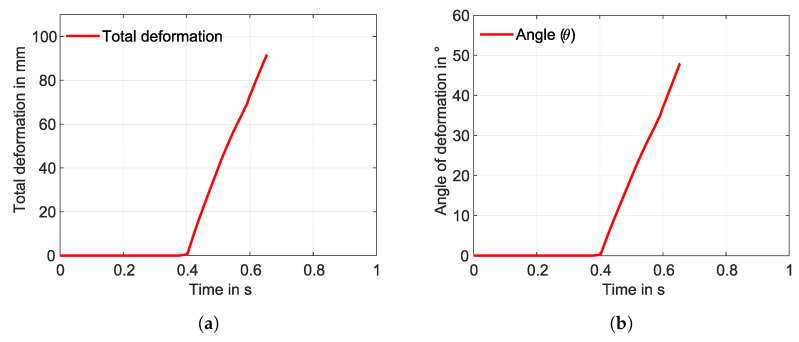
(**a**) Total deformation and (**b**) angle of deformation with respect to the time—WK model, straight SMA (simulation).

In the WK model, only one load step is used, with a maximum transformation strain of 3.5%. The same temperature boundary condition was applied to the SMA wire as that in the SA model, with the SMA wires in contact with the fixation element from the beginning. Upon transformation, a total deformation of 91.5 mm is observed (Figure 15a), with a deformation angle of 48.06°. The model did not obtain the convergence here as well, and upon observation, martensite residue can still be seen in Figure 15b. This indicates that the transformation is incomplete. The corresponding graphs for total deformation and angle of deformation are shown in Figure 16a and Figure 16b, respectively.

**Figure 17:**
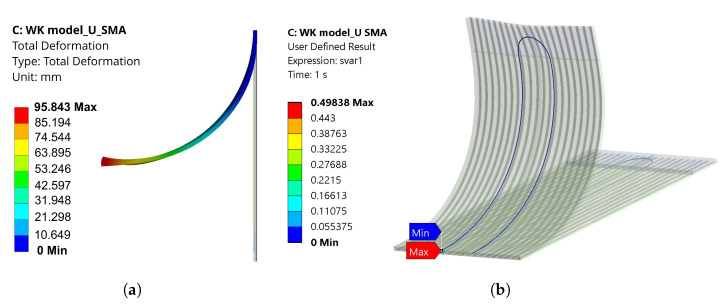
(**a**) Total deformation and (**b**) volume fraction from martensite (red colour) to austenite (blue colour) for U-profile SMA using the WK model.

**Figure 18:**
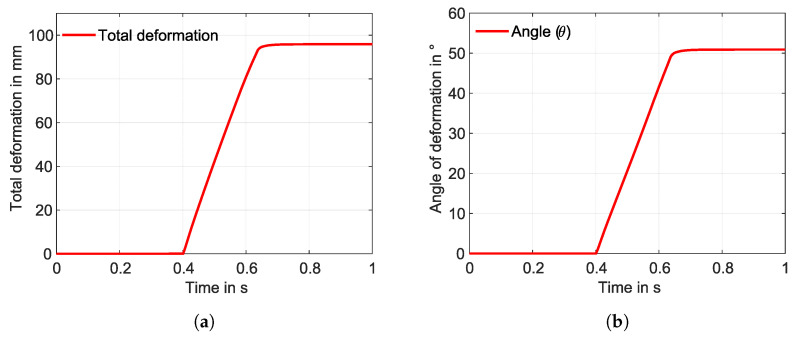
(**a**) Total deformation and (**b**) angle of deformation with respect to the time—WK model, U-profile SMA (simulation).

For the U-profile SMA wire simulated using a WK model, the same temperature boundary conditions were applied but without the need for the fixation element. Both ends of the SMA wire are fixed at one end, as shown in Figure 7b. In this case, a total deformation of 95.8 mm with a deformation angle of 50.89° is observed, which is shown in Figure 17a and Figure 18a,b. Here, the model converged completely with a complete transformation of the “U”-shaped SMA from martensite to austenite, as shown in Figure 17b.

### 3.2. Experiment

**Figure 19:**
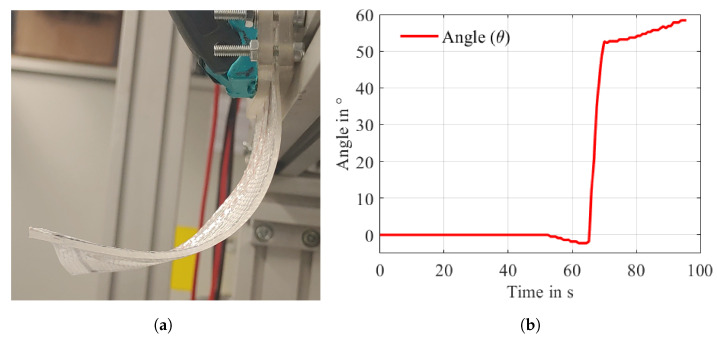
Experimental result for the IFRC. (**a**) IFRC after activation and (**b**) angle of deformation (only activation).

The activation of the SMA is performed using Joule heating. As it is tedious to have two straight SMA wires in one composite because of connectivity issues, the SMA wire is incorporated as a U-profile. No specific arrangements are required for the U-profile SMA, as both the ends of the SMA wire are fixed to the clamp. The activation of the IFRC, along with the graph depicting the deformation angle, is shown in Figure 19.

## 4. Discussion

Although both models had the same boundary conditions, mesh considerations, and material parameters, the total deformation is larger in the SA model than in the WK model for straight SMAs. The two models did not solve the problem entirely, which can be seen in the graphs (Figure 14a and Figure 16a). The SA model with the straight SMA wire showed a total deformation of 103.56 mm, whereas a total deformation of 91.5 mm was observed for the WK model. The transformation of the volume fraction from martensite to austenite is achieved completely in the SA model (Figure 13b), and an incomplete transformation is observed in the WK model (Figure 15b). This disparity led to the reduction of the total deformation observed. Upon observation, a few contact adversities exist between the fixation element and the other contact bodies for the straight SMA models. These adversities are linked in the form of small sliding between the SMA face and the fixation element, penetration of the fixation element into the matrix, and also the loss of contact between the SMA and the fixation element during iteration convergences. These adversities led to the high force convergence values, resulting in more difficult computation.

On the other hand, the WK model converged completely for the U-Profile SMA. A total deformation of 95.8 mm (Figure 17a) with a deformation angle of 50.89° (Figure 18b) is observed, and a complete transformation from martensite to austenite can be seen in Figure 17b. This complete convergence of the WK model for the U-profile SMA can support the statement of the contact adversities of straight SMAs. The maximum deformation angle obtained from the experiment is noticed to be 58.34°. The higher deformation angle in the experiment is linked to the overheating of the matrix surrounding the SMA, causing a slow increase in the deformation angle, as seen in the graph (Figure 19b). This slow increase of the deformation angle was not captured in the hyperelastic model acquainted elastomer, and therefore, a zero slope can be observed in the graph depicting the angle of deformation for the U-profile SMA (Figure 18b). This is because the temperature dependence of the elastomer is not considered. In the graph (Figure 19b), a negative angle is observed at the start of the austenite transformation. One of the reasons for this is that the IFRC structure is positioned as an overhanging structure and is activated with the help of Joule heating, in which the flow of voltage from a positive to a negative terminal induces heat, influencing the SMA to contract. This contraction occurs in the direction of the flow of heat, and so, at first, one side of the U-bend tries to contract, pulling the IFRC initially backwards. The amount of negative angle achieved is also linked to the tension created in the IFRC structure due to the fixation of the SMA on one end.

## 5. Conclusions

A comparison of the material models between the SA model and the WK model is made to validate the latter with respect to bending deformations. Initial comparisons for the simulation models were made by considering straight SMA profiles in the model composite structure. This consideration is due to the limitation of the SA model to generate deformations for curved or irregular SMA profiles. A U-profile SMA model is created and simulated using the WK model and is validated with the experiment. The straight SMA profile model and the U-profile SMA model are both validated using one experiment with the U-profile SMA IFRC structure. This is because of the nonfeasibility of fixing both ends of the SMA and activating it in parallel.

The validation results show comparable deformations for all the models, with little discrepancies. For the straight SMA wires, a 10% difference between the two material models is noticed, with the SA material model producing higher deformations than the WK model, and this can be linked to contact adversities. This can be related to the fact that the martensite to austenite transformation is observed to be incomplete due to poor convergence behaviour that occurred due to contact penetration, small sliding, and the loss of contacts during iterative convergences between the fixation element and the SMA-integrated composite structure. A fixation element is necessary to induce force onto the IFRC structure when the straight SMA contracts. On the other hand, the U-profile SMA has shown full convergence when using the WK model.

The deformation angle obtained in the experimental result showed 8° more deformation compared to the U-profile SMA simulated using the WK model. This difference is noticed due to the overheating of SMA during the experiment, causing the matrix to soften more at the curvature. This is not captured in the simulation, as the temperature dependence of the elastomer is neglected, thus creating a difference in the results. Further investigations and experimental procedures have to be developed to measure the deformations for straight SMA-integrated structures.

This paper investigates and validates the use of pre-stretching in the Woodworth and Kaliske model for irregularly shaped SMA wires. This model will be applied to future work, which consists mostly of the U-profile SMAs that will be used to obtain bend-twist coupling in the IFRC structures. Another way to obtain bend-twist coupling in the IFRC structures is to arrange the SMA wires in different positions pertaining to different directional deformations when activated. The WK model with pre-stretch allows for investigating the behaviour of such profile-embedded SMA structures.

## Figures and Tables

**Table 1:** Material parameters for silicone [41].

Model Parameter	Units	Value
$C_{10}$	Pa	770,623.97
$C_{20}$	Pa	−386,308.55
$C_{30}$	Pa	196,852.40
$d_{1}$	${Pa}^{-1}$	0
$d_{2}$	${Pa}^{-1}$	0
$d_{3}$	${Pa}^{-1}$	0

**Table 2:** Material parameters for glass fibre yarn [41].

Model Parameter	Units	Value
elastic modulus in x	GPa	5
elastic modulus in y	GPa	0.04
elastic modulus in z	GPa	0.04
Poisson’s ratio (xy)	-	0.22
Poisson’s ratio (yz)	-	0.22
Poisson’s ratio (xz)	-	0.22
shear modulus (xy)	GPa	0.04
shear modulus (yz)	GPa	0.02
shear modulus (xz)	GPa	0.04

**Table 3:** Material parameters for Nitinol wire in SA model [41].

Model Parameter	Units	Value at Room Temperature
austenite modulus	MPa	25,000
Poisson’s ratio	-	0.3
hardening parameter	MPa	610
reference temperature	°C	70
elastic limit	MPa	52
temperature scaling parameter	MPa/°C	7
maximum transformation strain	%	0.0355
martensite modulus	MPa	20,000
load dependency parameter	-	0

**Table 4:** Material parameters for Nitinol wire in the WK model [35].

Model Parameter	Units	Value at Room Temperature
shear modulus of austenite	MPa	9615.4
bulk modulus	MPa	20,833
hardening parameter	MPa	610
reference temperature	°C	70
elastic limit	MPa	52
temperature scaling parameter	MPa/°C	7
maximum transformation strain	%	0.0355
shear modulus of martensite	MPa	7692.3
initial martensite volume fraction	-	0.9
direction of pre-stretch (x, y, z)	-	(0,0,1)

## Data Availability

The data presented in this study are available on request from the corresponding author.

## Abbreviations

The following abbreviations are used in this manuscript:

FF	Functional Fatigue
GF	Glass Fibres
IFRC	Interactive Fibre Rubber Composites
MOSFET	Metal-Oxide-Semiconductor Field-Effect Transistor
Ni-Ti	Nickel-Titanium Alloy
OWSME	One-Way Shape Memory Effect
PDMS	Polydimethylsiloxane
PTFE	Polytetrafluoroethylene
PWM	Pulse Width Modulation
SMA	Shape Memory Alloy
SA	Souza and Auricchio
SME	Shape Memory Effect
TWSME	Two-Way Shape Memory Effect
TRIP	Transformation Induced Plasticity
TFP	Tailored Fibre Placement
UPFR	Uni-axial Pre-Strain Free Recovery
VARI	Vacuum Assisted Resin Infusion
WK	Woodworth and Kaliske

## References

[1] Hartl D.J., Lagoudas D.C. (2007). Aerospace applications of shape memory alloys. Proc. Inst. Mech. Eng. Part G J. Aerosp. Eng..

[2] Jani J.M., Leary M., Subic A., Gibson M.A. (2014). A review of shape memory alloy research, applications and opportunities. Mater. Des..

[3] Cianchetti M. (2013). Fundamentals on the Use of Shape Memory Alloys in Soft Robotics. Interdisciplinary Mechatronics.

[4] Petrini L., Migliavacca F. (2011). Biomedical Applications of Shape Memory Alloys. J. Metall..

[5] Kumar S., Peruvazhuthi S., Gopalakrishnan S. (2020). A half a decade timeline of shape memory alloys in modeling and applications. ISSS J. Micro Smart Syst..

[6] Van Humbeeck J. (2001). Shape Memory Alloys: A Material and a Technology. Adv. Eng. Mater..

[7] Lagoudas D. (2008). Shape Memory Alloys: Modeling and Engineering Applications.

[8] Chaudhari R., Vora J.J., Parikh D. (2021). A review on applications of nitinol shape memory alloy. Recent Advances in Mechanical Infrastructure.

[9] Alipour S., Taromian F., Ghomi E.R., Zare M., Singh S., Ramakrishna S. (2022). Nitinol: From historical milestones to functional properties and biomedical applications. Proc. Inst. Mech. Eng. Part H J. Eng. Med..

[10] Costanza G., Tata M.E. (2020). Shape Memory Alloys for Aerospace, Recent Developments, and New Applications: A Short Review. Materials.

[11] Budholiya S., Bhat A., Raj S.A., Hameed Sultan M.T., Md Shah A.U., A. Basri A. (2021). State of the Art Review about Bio-Inspired Design and Applications: An Aerospace Perspective. Appl. Sci..

[12] Kim H.I., Han M.W., Song S.H., Ahn S.H. (2016). Soft morphing hand driven by SMA tendon wire. Compos. Part B Eng..

[13] Liu C., Liao W. A Snake Robot Using Shape Memory Alloys. Proceedings of the 2004 IEEE International Conference on Robotics and Biomimetics.

[14] Sugiyama Y., Hirai S. (2006). Crawling and Jumping by a Deformable Robot. Int. J. Robot. Res..

[15] Hao L.H., Park S.H., Park J.O. Shape Memory Alloy based Flower Robot. Proceedings of the 39th International Symposium on Robotics.

[16] Wang Z., Hang G., Li J., Wang Y., Xiao K. (2008). A micro-robot fish with embedded SMA wire actuated flexible biomimetic fin. Sens. Actuators A Phys..

[17] Chen X., Shigemune H., Sawada H. An Untethered Bionic Robotic Fish Using SMA Actuators. Proceedings of the 2020 IEEE International Conference on Mechatronics and Automation (ICMA).

[18] Coral W., Rossi C., Curet O.M., Castro D. (2018). Design and assessment of a flexible fish robot actuated by shape memory alloys. Bioinspiration Biomimetics.

[19] Xu L., Wagner R., Liu S., He Q., Li T., Pan W., Feng Y., Feng H., Meng Q., Zou X. (2022). Locomotion of an untethered, worm-inspired soft robot driven by a shape-memory alloy skeleton. Sci. Rep..

[20] Maeno T., Hino T. Miniature five-fingered robot hand driven by shape memory alloy actuators. Proceedings of the IASTED International Conference on Robotics and Applications.

[21] Lee J.H., Chung Y., Rodrigue H. (2019). Long Shape Memory Alloy Tendon-based Soft Robotic Actuators and Implementation as a Soft Gripper. Sci. Rep..

[22] Frémond M., Rocca E. (2009). A model for shape memory alloys with the possibility of voids. Discret. Contin. Dyn. Syst..

[23] Colli P., Frémond M., Rocca E., Shirakawa K. (2006). Attractors for a Three-Dimensional ThermoMechanical Model of Shape Memory Alloys. Chin. Ann. Math. Ser. B.

[24] Falk F., Konopka P. (1990). Three-dimensional Landau theory describing the martensitic phase transformation of shape-memory alloys. J. Phys. Condens. Matter.

[25] Colli P., Sprekels J. (1993). Positivity of temperature in the general Frémond model for shape memory alloys. Contin. Mech. Thermodyn..

[26] Brinson L. (1993). One-Dimensional Constitutive Behavior of Shape Memory Alloys: Thermomechanical Derivation with Non-Constant Material Functions and Redefined Martensite Internal Variable. J. Intell. Mater. Syst. Struct..

[27] Dunić V., Busarac N., Slavković V., Slavković R. Thermo-mechanical numerical analysis of stent unit cell. Proceedings of the 2015 IEEE 15th International Conference on Bioinformatics and Bioengineering (BIBE).

[28] Gédouin P.A., Pino L., Bourgeot J.M., Delaleau E., Chirani S.A., Calloch S. Thermo-mechanical modelling of spring-based SMA actuators. Proceedings of the 2016 11th France-Japan & 9th Europe-Asia Congress on Mechatronics (MECATRONICS)/17th International Conference on Research and Education in Mechatronics (REM).

[29] Sedlák P., Frost M., Benešová B., Ben Zineb T., Šittner P. (2012). Thermomechanical model for NiTi-based shape memory alloys including R-phase and material anisotropy under multi-axial loadings. Int. J. Plast..

[30] Choudhry S., Yoon J.W. (2004). A General Thermo-Mechanical Shape Memory Alloy Model: Formulation and Applications. AIP Conf. Proc..

[31] Auricchio F., Petrini L. (2002). Improvements and algorithmical considerations on a recent three-dimensional model describing stress-induced solid phase transformations. Int. J. Numer. Methods Eng..

[32] Souza A.C., Mamiya E.N., Zouain N. (1998). Three-dimensional model for solids undergoing stress-induced phase transformations. Eur. J. Mech.-A/Solids.

[33] Grandi D., Stefanelli U. (2014). The Souza-Auricchio model for shape-memory alloys. Discret. Contin. Dyn. Syst.-Ser. S.

[34] Woodworth L.A., Kaliske M. (2022). A temperature dependent constitutive model for functional fatigue in shape memory alloys. Mech. Mater..

[35] Woodworth L.A., Lohse F., Kopelmann K., Cherif C., Kaliske M. (2022). Development of a constitutive model considering functional fatigue and pre-stretch in shape memory alloy wires. Int. J. Solids Struct..

[36] Mizzi L., Spaggiari A., Dragoni E. (2020). Design of shape memory alloy sandwich actuators: An analytical and numerical modelling approach. Smart Mater. Struct..

[37] Song J.J., Chen Q., Naguib H.E. (2016). Constitutive modeling and experimental validation of the thermo-mechanical response of a shape memory composite containing shape memory alloy fibers and shape memory polymer matrix. J. Intell. Mater. Syst. Struct..

[38] Wang X. (2002). Shape memory alloy volume fraction of pre-stretched shape memory alloy wire-reinforced composites for structural damage repair. Smart Mater. Struct..

[39] Cherif C. (2016). Textile Materials for Lightweight Constructions.

[40] Kyosev Y. (2014). Braiding Technology for Textiles: Principles, Design and Processes.

[41] Lohse F., Kopelmann K., Grellmann H., Ashir M., Gereke T., Häntzsche E., Sennewald C., Cherif C. (2022). Experimental and Numerical Analysis of the Deformation Behavior of Adaptive Fiber-Rubber Composites with Integrated Shape Memory Alloys. Materials.

[42] Mersch J., Bruns M., Nocke A., Cherif C., Gerlach G. (2021). High-Displacement, Fiber-Reinforced Shape Memory Alloy Soft Actuator with Integrated Sensors and Its Equivalent Network Model. Adv. Intell. Syst..

[43] Simon J., Hamila N., Binetruy C., Comas-Cardona S., Masseteau B. (2022). Design and numerical modelling strategy to form Tailored Fibre Placement preforms: Application to the tetrahedral part with orthotropic final configuration. Compos. Part A Appl. Sci. Manuf..

[44] Annadata A., Endesfelder A., Koenigsdorff M., Mersch J., Gereke T., Zimmermann M., Cherif C. Evaluation of Bend-Twist Coupling in Shape Memory Alloy Integrated Fiber Rubber Composites. Proceedings of the 9th ECCOMAS Thematic Conference on the Mechanical Response of Composites (COMPOSITES 2023).

[45] Yeoh O.H. (1993). Some Forms of the Strain Energy Function for Rubber. Rubber Chem. Technol..

[46] Lohse F., Annadata A.R., Häntzsche E., Gereke T., Trümper W., Cherif C. (2022). Hinged Adaptive Fiber-Rubber Composites Driven by Shape Memory Alloys—Development and Simulation. Materials.

[47] Stachiv I., Alarcon E., Lamac M. (2021). Shape Memory Alloys and Polymers for MEMS/NEMS Applications: Review on Recent Findings and Challenges in Design, Preparation, and Characterization. Metals.

[48] Coda A., Cadelli A., Manjeri M., Sczerzenie F. (2015). Uniaxial Pre-strain and Free Recovery (UPFR) as a Flexible Technique for Nitinol Characterization. Shape Mem. Superelasticity.

